# New anti–coagulant therapies set to revitalise clinical haemotology practice 

**Published:** 2010-12

**Authors:** AALBERS J, WAGENAAR P, KLUG E

**Affiliations:** Special Assignment Editor; Johannesburg correspondent; Sunninghill Hospital, Johannesburg

‘For all patients with stents undergoing surgery or other revascularisation procedures, do not stop the low–dose aspirin of 81 mg. If you have to, withdraw the thienopyridine and reintroduce as soon as possible’

– Dr Eric Klug

Clinical and laboratory–based haematologists are likely to experience more requests for advice and support from their colleagues in cardiology, orthopaedics and neurology as the impact of newly registered anti–coagulant therapies unfolds. This is because the new anti–thrombotic and anti–platelet agents will need to be understood on the basis of their individual attributes and not as inter–changeable drugs within their respective class. Also, usage of the new factor Xa inhibitors such as rivaroxaban, and direct thrombin inhibitors such as dabigatran, while not requiring monitoring for routine clinical practice, have different effects on standard coagulation tests which may require expert interpretation prior to surgery or percutaneous coronary interventions (PCI), or to assess compliance with medication or suspected over–dosing.

## A practical approach to new anti-platelet agents

***Dr Eric Klug, Sunninghill Hospital, Johannesburg***

‘There is an association between bleeding events and long–term mortality; the occurrence of a major bleeding event is associated with a continued higher risk of death at one year’, Dr Eric Klug noted. The key risk factors associated with bleeding risk include impaired renal function, older age, female gender, preceding anaemia and heart failure.^1^ ‘This list of course also overlaps critically with the increased risk of ischaemic events’, Dr Klug pointed out.

The choice of antiplatelet agents has expanded from aspirin with its well–accepted role in primary and secondary prevention of cardiovascular and cerebrovascular events to include new agents such as clopidogrel, prasugrel and ticagrelor.

‘Concern has been expressed about prior aspirin use and outcomes in acute coronary syndromes (ACS). This was recently evaluated in data from 60 000 patients with ACS who participated in myocardial infarction clinical trials.^2^ The increased mortality found in patients taking aspirin prior to presentation with an ACS was related to the inherent higher risk of these patients rather than to any adverse effects of the aspirin’, Dr Klug advised. With regard to preventing post–stent thrombosis, dual anti–platelet therapy (DAPT) is the gold standard; aspirin is used together with a thienopyridine such as clopidogrel, prasugrel or ticagrelor.

‘These agents are very effective, but the problem with regard to cardiovascular events after the stenting procedure is related to withdrawal of dual anti–platelet therapy. In the largest study of drug–eluting stent–associated thrombosis,^3^ a higher incidence of stent thrombosis occurred following the discontinuation of both aspirin and clopidogrel (or other thienopyridine) within a short period, relating to the drug discontinuation’, Dr Klug said.

‘It is clear that we should always try to maintain aspirin therapy at least at 81 mg/day during and post stenting, and also where possible delay non–cardiac surgery for at least three months after the PCI. In the latter case, a risk remains, but the delay of surgery is beneficial’, Dr Klug added. He noted that a tapered withdrawal of the selected anti–platelet therapy to avoid the so–called rebound phenomenon is not required and the clopidogrel can be stopped abruptly.^4^

The anti–platelet action of clopidogrel is lessened in patients with homozygous genetic variations of the CYP2C 19 gene, which can be significant in high–risk patients. ‘This is also true for prasugrel as both are pro–drugs, requiring conversion via the cytochrome P450 enzyme. However, as cardiologists, we cannot wait for genetic studies; nor has it been shown that doing routine genetic testing improves clinical outcomes. It is for this reason that the FDA has taken the decision to warn clinicians about this possibility, but not mandate genetic testing’, Dr Klug said.

Evidence for the additional value of prasugrel compared to clopidogrel in achieving a greater reduction of stent thrombosis, cardiovascular death, myocardial infarction and stroke in patients with ACS undergoing PCI comes chiefly from the TRITON TIMI 38 trial.^5^ ‘The benefits of prasugrel over clopidogrel unfortunately occurred with an increased risk of major bleeding, including fatal bleeding’, Dr Klug pointed out. ‘Subsequent clinical use has helped define a group of patients who should not be given the more potent thienopyridine (prasugrel) as being those older than 75 years, or with a history of stroke/transient ischaemic attack (TIA), or with a low body weight, less than 60 kg.’ Prasugrel can however be used for NSTEMI patients and for STEMI patients treated with PCI.

Dr Klug presented insights on the newer oral anti–platelet agents such as ticagrelor, which is more effective than clopidogrel without the penalty of increased bleeding rates. Dyspnoea is however common, but seems to be self–limiting and results in less than 1% of patients discontinuing the drug. New reversible P2y(12) receptor inhibitors such as elinogrel and cangrelor are currently in clinical development trials.

‘Clearly, the &quot;one size fits all&quot; and &quot;one mechanism fits all&quot; usage of anti–platelet agents reflects limitations of current evidence and of our lack of understanding. This will change in the near future’, Dr Klug concluded.

‘Evidence–based medicine provides the structure; in the care of the individual patient clinical insight and judgement are always paramount’

– Dr Eric Klug

## Anti-coagulation in atrial fibrillation

***Prof Lord Kakkar, London, UK***

‘Anti–coagulation to prevent thrombo–embolic stroke is vital, as about one–sixth of all strokes are due to pre–existing atrial fibrillation’, Prof Kakkar stressed at the outset of his presentation. While his talk concentrated on the role of anti–coagulation to prevent thrombo–embolic stroke, Prof Kakkar noted that co–morbid conditions that heighten the potential risk of stroke in atrial fibrillation patients, such as hypertension, heart failure, arterial disease and diabetes, also require treatment with the increasingly effective therapies that are now available.

Warfarin became the standard of care from the early studies of vitamin K antagonists, which compared warfarin use to placebo and showed a 70% reduction in the frequency and morbidity of strokes. ‘Warfarin is also better than aspirin in preventing thrombo–embolic stroke; while full–dose warfarin, reaching INR targets of between 2 and 4, is better than low–dose warfarin’, Prof Kakkar noted. ‘Despite warfarin’s known benefits, if we look at the atrial fibrillation (AF) studies in some 11 000 patients in the USA from either clinical trials or registries, only 50 to 60% of patients are treated with this agent. So large numbers of patients who could derive benefit are not receiving medication to reduce their stroke risk.’

With regard to novel anti–coagulant drugs, a number are under development, and target different factors in the coagulation cascade, such as activated factor X or activated factor II (thrombin). As there are limited data from prospective phase III randomised clinical trials of these new agents, Kakkar concentrated on available results of rivaroxaban and the evidence for dabigatran, an orally active direct inhibitor of thrombin.

The ROCKET study of rivaroxaban in atrial fibrillation has been presented at the American Heart Association meeting. It is a large study of 14 000 patients randomised to receive either warfarin to a target INR of 2.5 mg or rivaroxaban 20 mg once daily or 15 mg/day for patients with moderate renal impairment. It showed non–inferiority of rivaroxaban to warfarin in AF.

Apixaban, a second orally active Xa inhibitor is being studied in the ARISTOTLE study in AF, with dosages of 5 mg bid apixaban versus warfarin. The results of the AVERROES study on patients with atrial fibrillation who are unsuitable for a vitamin K antagonist was announced at the European Society of Cardiology in September 2010. Apixaban was shown to be significantly better than aspirin in reducing the frequency of stroke or systemic embolic events in this study. ‘This is certainly interesting data as there was no significant increase in major bleeding complications with apixaban versus aspirin’, Prof Kakkar pointed out. Recently an ACS trial with apixiban added to aspirin has been stopped early because of increased bleeding in the combination arm compared to aspirin alone.

Results of the RE–LY study of two doses of dabigatran (110 or 150 mg bid) compared to warfarin dosages aimed at an INR of 2.5 in at–risk atrial fibrillation patients have been published. ‘These results are quite striking, with the lower dose of dabigatran being equal to warfarin (1.53 event rate vs 1.69) in reducing the rate of stroke and systemic embolic events. The 150–mg dabigatran dose BD not only achieved non–inferiority, but was superior to warfarin in reducing events. Both doses of dabigatran were associated with a lower incidence of haemorrhagic stroke than seen with warfarin. This is certainly the most striking of the positive results for dabigatran, as this is a most feared bleeding event’, Dr Kakkar stressed.

Importantly, dabigatran performed well throughout the twoand– a–half years of follow up across all INR ranges of warfarin treatment and regardless of whether patients were exposed previously to vitamin K antagonists or were vitamin K naïve.

## Rivaroxaban – the latest Einstein results

***Prof Harry Buller, Amsterdam, Netherlands***

The results of several Einstein studies provide the clinician with some valuable insights into the utility of rivaroxaban as a treatment option for long–term anticoagulation. Prof Buller reviewed these findings and their implications at the Southern African Society of Thrombosis and Haemostasis conference on 31 October 2010.

In the initial treatment of venous thrombo–embolism (VTE), there are a number of treatment options, of which low–molecularweight heparin is the most frequently used. However, vitamin K antagonists have been the only choice for extended treatment, i.e. three to six months or longer. The question of how long to continue treatment is now being reconsidered and there is a strong move in the USA and Canada towards continuing treatment indefinitely. ‘Our understanding of anticoagulation at the molecular level also means that the number of anticoagulants available to us has increased dramatically in recent years – and there are many more still in development’, said Prof Buller. He cautioned against viewing the new drugs by class, underscoring that each needed to be viewed individually.

Three Einstein studies recently evaluated rivaroxaban relative to enoxaparin followed by a vitamin K antagonist, with a view to showing non–inferiority. The Einstein DVT (deep–vein thrombosis) and PE (pulmonary embolism) studies were 30–day observational studies that evaluated rivaroxaban (15 mg twice daily or 20 mg once daily). The Einstein Extension study looked at patients with combined DVT and PE.

The Einstein DVT study’s main efficacy measure was the prevention of recurrences, while safety was assessed in terms of major and clinically relevant non–major bleeding. The findings in respect of first symptomatic recurrence of VTE were 2.1% for rivaroxaban and 3% for enoxaparin plus warfarin. For recurrent DVT, they were 0.8% for rivaroxaban and 1.6% for enoxaparin plus warfarin. ‘There was therefore strong evidence that rivaroxaban – a single drug given in a fixed dose – is at least as good as the comparator’, continued Prof Buller. ‘When primary efficacy outcomes were evaluated by subgroup, there was also a tendency in favour of rivaroxaban, with variables such as gender, body weight and creatinine clearance making no difference to the drug’s efficacy.’

When it came to safety, the results were similar, suggesting that rivaroxaban is as safe as it is effective. Once again, the subgroup analysis showed no outliers. Findings in respect of key secondary outcomes were as follows: net clinical benefit – 2.9% for rivaroxaban vs 4.2% for enoxaparin plus warfarin; total mortality – 2.2 vs 2.9%; cardiovascular events – 0.7 vs 0.8% and liver complications – 0.1 vs 0.2%, respectively.

Summarising, Prof Buller concluded that the findings suggest that rivaroxaban offers a single–drug approach for both acute and long–term anticoagulation. It is non–inferior to enoxaparin plus warfarin in respect of efficacy and safety, works consistently across subgroups, and has no associated liver toxicity.

The Einstein Extension study evaluated rivaroxaban against placebo. Patients were treated for an average of 249 days. Symptomatic recurrent VTE occurred in 7.1% of patients on placebo vs 1.3% on rivaroxaban. The figures were 5.2% and 0.8%, respectively, for recurrent DVT. The principal safety outcome – recurrence of major bleeding – was of course, 0% for placebo but only 0.7% for rivaroxaban.

Clinically relevant non–major bleeding did occur, however, in 5.4% of those treated with rivaroxaban, versus 1.2% of those on placebo. This was a statistically significant finding. Prof Buller underscored, therefore, that it is important to bear in mind that rivaroxaban’s long–term efficacy advantage does come at a cost.

‘Rivaroxaban brings about an 82% relative risk reduction in the recurrence of VTE, he concluded, and it’s a simpler treatment option. What is important is the need to consider very carefully whether continued anticoagulation is indeed indicated.’

## Clinical trials and bleeding – making sense of the results

***Prof Sylvia Haas, Technical University, Munich, Germany***

Prof Haas spotlighted the many confounders that play a role in the widely varying rates of major bleeding seen in hip and knee arthroplasty trials. Among the factors that need to be considered are: definition of what constitutes major bleeding; timing of assessment of bleeding, whether pre or post surgery; timing of administration of anticoagulants including the comparator agent (enoxaparin), whether pre or early/late post surgery; dose and duration of anticoagulation; and the collection of bleeding data and adjudication of events. ‘And then there’s also the play of chance’, she added.

There are therefore uncertainties and imponderabilities when comparing bleeding rates across trials. When one looks at the phase III trials of hip and knee replacements, one sees varying definitions between trials and hence variance in the bleeding rates from trial to trial. The bleeding rates in RE–NOVATE 1 and 2 (which evaluated dabigatran versus enoxaparin in total hip replacement) differed because the comparator bleeding results with enoxaparin at the same dose in each trial was different. The adjudication of venograms may be different between trials as well, and are only standardised within a particular trial; therefore across–trial comparisons are often not helpful or accurate.

She underscored that it was therefore important to only compare what can be directly compared. A study by Huisman et al. (submitted for publication) pooled the dabigatran trials, excluding RENOVATE 2, and proved non–inferiority of dabigatran’s efficacy relative to that of enoxaparin, in respect of the primary endpoints of symptomatic VTE and all–cause mortality. Similarly, in a pooled study of the RECORD trials, without RECORD 2, that evaluated rivaroxiban relative to enoxaparin with the same endpoints, it showed the clear–cut superiority of rivaroxaban.

‘One RECORD trial on its own met the primary endpoint criteria of superiority, but with the added value of the other two trials, there was a highly significant result in favour of rivaroxaban’, Prof Haas said. She warned that meta–analyses don’t necessarily provide proof but are primarily hypothesis generating, as those undertaking them usually don’t have access to the source data.

Prof Haas and her team did, however, have access to the source data when they undertook a pooled analysis of RECORD 1–4 (submitted for publication). ‘The authors have been able to include almost all (98%) patient data’, she said. ‘The efficacy results were highly statistically significant for rivaroxaban versus enoxaparin. While the combined results initially disfavoured rivaroxaban, when it came to major bleeding, the differences disappeared when we looked at only the period when all patients were on active study medication. There was also no statistical difference in bleeding rates using this approach, regardless of whether we applied the ISTH or EMEA definitions of major bleeding’, she added.

Concluding, she gave the following take-home messages:

- Objective assessment of bleeding in patients undergoing surgery is a challenge, as there are just too many factors influencing the rates of major bleeding.
- Meta–analyses are helpful for hypothesis generation, but much less so for providing confirmed results.
- The increasing number of meta–analyses does not help solve the problem.
- Well–designed non–interventional studies could be more important to assess bleeding rates in the real world.

## Monitoring of direct coagulation inhibitors – the way forward?

***Prof Sylvia Haas, Technical University, Munich, Germany***

Addressing what users of these new agents need to know in the clinical setting, Prof Haas pointed out that indirect factor Xa inhibitors such as fondaparinux interact with free factor Xa and do not alter prothrombin time (PT) measurements and interpretation thereof. However, the direct factor Xa inhibitors rivaroxaban and apixaban directly interact with the factor Xa molecule and the prothrombinase complex, thereby influencing prothrombin time and related measurements. ‘They interfere with INR reliability and if you do this test and get INR values of between 2 and 4, you could think that the patient is fully anti–coagulated, but this is not necessarily so’, she pointed out.

For rivaroxaban, PT is the most sensitive anti–coagulation test as there is a concentration–dependent prolongation, but interpretation depends on the specific reagents being used. Applying a standard calibration curve to the PT test results allows for correlation with the plasma concentrations of rivaroxaban.^6^

Dabigatran, the direct thrombin inhibitor weakly influences PT and strongly affects the partial thromboplastin time (PTT), while only weakly affecting the INR measurement and interpretation. ‘A new test, hemoclot, which is a diluted thrombin test, offers promise in assessing dabigatran anti–coagulation, but is not yet available on the market.

‘The discussion is, however, ongoing with regard to the most useful tests and we are encouraging manufacturers to develop appropriate safety tests for their therapies’, Prof Haas stressed. Currently the chromagenic assays will give the most accurate reflection of rivaroxaban or dabigatran levels and anti–coagulant status’, Prof Haas pointed out.^7^ At a pragmatic level, a pocket card with the t–max and t–half–life of these agents can be useful to assess the coagulation state if the time and dose of last medication is known.

**Monitoring of direct coagulation inhibitors**

- There is no need for routine monitoring, as a standard dose of the new anti–coagulants is used.
- New anti-coagulant agents affect conventional clotting tests.
- Do not routinely measure PTT/PT when using these agents.
- Use the specific tests, as advocated by the manufacturers, for suspected over– or under–dosing.

## D-dimers – how do we use this test clinically?

***Prof Harry Buller, Amsterdam, Netherlands***

It is essential to assess the clinical probability of either deepvein thrombosis or pulmonary embolism in an individual patient before initiating and interpreting a D–dimer test, Prof Buller advised. ‘The D–dimer is a valuable test to identify fibrin–derived products. Although the ELISA version is highly sensitive, clinical urgency does not often allow time for this test. Instead, latex–based tests are used; they are sensitive and rapid, but have a lower specificity.’

In an evaluation of published trials that determined the prevalence of DVT using clinical prediction rules for the diagnosis of DVT, Wells and colleagues8 determined the likelihood ratios of DVT in low, moderate and high clinical probability–assessed groups. ‘Patients with a low clinical probability of DVT, using the Wells predictive rule, and a subsequent negative D–dimer test can be excluded from ultrasound evaluation’, Prof Buller noted. ‘Patients in the moderate–risk category with a raised D–dimer value should undergo compression ultrasound for confirmation of DVT. In high–risk VTE patients, one should rather ignore the need to do the D–dimer test and go directly to compression ultrasound’, Prof Buller advised.

A useful diagnostic management protocol to determine the probability of pulmonary embolism has been developed and prospectively tested by the so–called Christopher study group.^9^ Prof Buller commented; ‘this option is attractive in that 30% of patients with clinically suspected pulmonary embolism can be excluded by using the clinical probability score in combination with a normal D–dime test result. In all other patients, computed tomography (CT) scans effectively rule out pulmonary embolism without using other imaging tests. In fact, there was only a 1.3% incidence of VTE in the subsequent three months in patients with a negative CT scan.’

In conclusion, Prof Buller referred to new data indicating that the cut–off values for D–dimer tests may well be higher in the elderly, and a prospective study is currently underway to determine normal values in this patient population. With regard to using D–dimer tests to determine optional length of anticoagulation therapy, Prof Buller noted that the normal D–dimer test has little value in this situation, as the sensitivity is very low (43%). ‘We would harm the majority of patients if we used this parameter and stopped anti–coagulation therapy too early’, he added. In cancer patients, D–dimer tests can be predictive and prognostic to some extent, but this is still at a research stage.

## Thrombolytics in stroke patients

***Dr Jody Pearl, Sunninghill Hospital, Johannesburg***

Facing a lack of therapeutic innovation in the treatment of stroke, Dr Pearl referred to the successful stroke intervention protocol set up at the Vergelegen Medi–Clinic, Somerset West. This unit provides a 24–hour intervention service similar to the acute stroke units in London, which have successfully intervened to significantly drop the mortality from stroke. The concept is that ‘time is brain’ and the patient needs to get to the appropriate centre quickly, where neurologists and interventional radiologists or cardiologists are on call and available to provide a 24–hour support service. Fibrinolytic therapy and/or percutaneous thrombosis aspiration devices are the current options available, depending on the patient characteristics. This approach should be adopted more widely in South Africa.

## Minimally invasive surgery for carotid disease – where are we?

***Prof Talib Abdul Carrim, University of Kwazulu-Natal, Durban***

While the debate continues as to whether carotid artery stenting (CAS) or carotid endarterectomy (CAE) is the preferred strategy for carotid disease, two issues remain: (1) what are the indications in 2010 for each procedure; and (2) is treating asymptomatic patients with significant stenoses – CAS – unethical as the procedure itself may be associated with a 6.9% increased risk. Prof Carrim indicated that some clarity was emerging as to which procedure best suits which patient and when one should submit a patient.

After the CREST study,10 which showed no difference in overall short– and long–term outcomes of these two techniques, new analyses are beginning to identify appropriate patient selection. In CREST, it was shown that outcomes with CAS were better than CEA for patients less than 70 years of age. In two recent meta–analyses,^11^,^12^ CEA was shown to be better than CAS; but both reviews acknowledge that the treatment strategy chosen should best meet the individual patient’s risk.

‘At this juncture we can conclude that CAS is not indicated in the elderly, in those with disease situated in difficult–to–reach sites of the carotid artery, and those with echolucent plaques that are more liable to rupture.

**Summary of CAS and CEA characteristics**

CAS

- increased peri-operative stroke incidence
- higher restenosis rates
- poor outcomes in those older than 70 years
- higher death rates in the elderly and in high–risk sites (difficult to reach and echolucent plague)
- longer term outcome equal to CEA

CEA

- remains the gold standard
- periprocedural MI and cranial nerve injury higher than in CAS
- better for patients with unstable plaque
